# An advanced fire detection system for assisting visually challenged people using recurrent neural network and sea-horse optimizer algorithm

**DOI:** 10.1038/s41598-025-91829-9

**Published:** 2025-07-01

**Authors:** Fahd N. Al-Wesabi, Abeer A. K. Alharbi, Ishfaq Yaseen

**Affiliations:** 1 Department of Computer Science, Applied College at Mahayil, King Khalid University, Abha, Saudi Arabia; 2 Department of Information Systems, College of Computer and Information Sciences, Imam Mohammad Ibn Saud Islamic University (IMSIU), Riyadh, 11432 Saudi Arabia; 3 Department of Computer and Self Development, Preparatory Year Deanship, Prince Sattam bin Abdulaziz University, AlKharj, Saudi Arabia; 4 King Salman Center for Disability Research, Riyadh, 11614 Saudi Arabia

**Keywords:** Fire detection, Attention mechanism, Sea-horse optimizer, Sobel filtering, Feature extraction, Computational biology and bioinformatics, Mathematics and computing

## Abstract

The developing elderly population undergoes a high level of eyesight and mental impairment, which frequently results in a defeat of independence. That kind of person should do vital daily tasks like heating and cooking, with methods and devices intended for visually unaffected persons, which does not consider the requirements of people with blind and intellectual impairment. Innovative technology needs the proper techniques for perceiving fires as rapidly as possible to avert damages. Initial fire recognition and notification models deliver fire inhibition and protection information to visually challenged individuals in an emergency if a fire happens indoors. Using an early fire recognition and warning model for blind individuals can decrease the number of victims, the number of losses, and, most essentially, early deaths. Recently, the growth of the fire recognition approach using artificial intelligence (AI) has advanced in helping blind people. This manuscript presents a Smart Fire Detection System for Assisting the Blind Using Attention Mechanism-Driven Recurrent Neural Network and Seahorse Optimizer Algorithm (SFDAB-ARNNSHO). The main intention of the SFDAB-ARNNSHO method is to detect and classify fire for blind people. To achieve this, the proposed SFDAB-ARNNSHO model performs image pre-processing by utilizing the sobel filtering (SF) model to remove noise in input data. Furthermore, the fusion of feature extraction comprises three methods, EfficientNetB7, CapsNet, and ShuffleNetV2. Furthermore, the SFDAB-ARNNSHO model performs fire detection and classification using stacked two-layer bidirectional long short-term memory with attention mechanism (SBiLSTM-AM) technique. Finally, the parameter tuning of the SBiLSTM-AM method is accomplished by implementing the seahorse optimizer (SHO) technique. The simulation validation of the SFDAB-ARNNSHO methodology is examined under the fire detection dataset, and the outcomes are measured using various measures. The performance validation of the SFDAB-ARNNSHO methodology portrayed a superior accuracy value of 99.30% over existing models under diverse measures.

## Introduction

Disorders in the visual system are the reason for blindness and visual impairment, which can prevent persons from performing housework and hinder their travel, work, studies, and participation in sports^1^. The global population is rising quickly, and an increasing number of individuals are alive well into old age, increasing the number of visually impaired and blind (VIB) people. Blind and elderly individuals face various difficulties in executing everyday actions, including environmental awareness, housework, travelling, work, and because of physical and sports practice, cognitive decline, and sensory^2^. This condition wants significant attention because the amount of individuals with visual impairment is ready to rise highly in the future decades. These numbers are mainly crucial in advanced countries as progress in medical care methods has allowed a long life. Various solutions have been found to address such difficulties, and assistive and software technology remains advanced to aid visually impaired individuals^3^. Applying an early fire recognition and notification method for VIB persons can decrease the count of victims, property damage, and, most significantly, the number of premature deaths. The VIB should take extra precautions regarding safety and health, particularly fire safety^4^. Some assistive gadgets offer improved protection to blind people compared to self-initiated life safety procedures. Efficient fire detection is unique to VIB individuals and can assist in maintaining an individual’s life in a fire emergency. Initial fire recognition is vital, as it directly influences human safety and the environment^5^. An entirely automatic fire recognition and notification method was advanced for BVI individuals to offer safety information in emergency and fire prevention^6^. Conventionally, fires have been recognized utilizing sensory methods that identify alterations in temperature or smoke in indoor surroundings. Nearly every fire detection method nowadays has built-in sensors, and thus, the methods are substantially based on spatial dispersion and reliable sensors^7^. Large space coverage in outdoor or indoor surroundings is impractical in a sensor-based fire recognition method due to the necessity for nearby regular distribution sensors; therefore, such a method has a higher false alarm rate^8^. AI-based fire recognition is the prevailing method to identify flames and notify building occupants in diverse indoor surroundings. It is highly distinctive, is not based on shape, size, or colour, and is firm to lighting changes^9^. To overwhelm the problematic machine learning (ML), fire detection, deep learning (DL) based computer vision methods are applied to define fire detection when outdoor or indoor fire detection methods cannot. Researchers and scientists select ML and DL methods; meanwhile, they present enhanced predictions compared to other methods^10^. Both methods are applied to ensure automated feature extraction by training complex features to obtain more informative data demonstration.

This manuscript presents a Smart Fire Detection System for Assisting the Blind Using Attention Mechanism-Driven Recurrent Neural Network and Seahorse Optimizer Algorithm (SFDAB-ARNNSHO). The main intention of the SFDAB-ARNNSHO method is to detect and classify fire for blind people. To achieve this, the proposed SFDAB-ARNNSHO model performs image pre-processing by utilizing the sobel filtering (SF) model to remove noise in input data. Furthermore, the fusion of feature extraction comprises three methods, EfficientNetB7, CapsNet, and ShuffleNetV2. Furthermore, the SFDAB-ARNNSHO model performs fire detection and classification using stacked two-layer bidirectional long short-term memory with attention mechanism (SBiLSTM-AM) technique. Finally, the parameter tuning of the SBiLSTM-AM method is accomplished by implementing the seahorse optimizer (SHO) technique. The simulation validation of the SFDAB-ARNNSHO methodology is examined under the fire detection dataset, and the outcomes are measured using various measures. The key contribution of the SFDAB-ARNNSHO methodology is listed below.

- The SFDAB-ARNNSHO model integrates SF-based pre-processing to improve image quality by detecting edges, significantly improving fire detection accuracy. This step ensures improved feature extraction by emphasizing crucial boundaries and structures in the images. By refining image details, the model attains more precise classification and detection of fire events.
- The SFDAB-ARNNSHO method incorporates feature extraction from three powerful methods, EfficientNetB7, CapsNet, and ShuffleNetV2, utilizing their merits to improve feature learning. This integration allows the model to capture both global and local features effectively. By harnessing these techniques, the model attains a more robust and comprehensive understanding of the input data for fire detection.
- The SFDAB-ARNNSHO approach employs the SBiLSTM-AM method to improve fire detection and classification. This architecture processes sequential data, capturing temporal dependencies and improving model performance. The attention mechanism additionally optimizes focus on critical features, ensuring accurate classification.
- The SFDAB-ARNNSHO technique utilizes the SHO methodology to optimize the model’s performance by efficiently tuning hyperparameters. It also enhances the fire detection system’s accuracy and efficiency. By fine-tuning the parameters, SHO ensures that the model operates at its best performance for real-world applications.
- The novelty of the SFDAB-ARNNSHO approach is in integrating advanced feature extraction methods, such as EfficientNetB7, CapsNet, and ShuffleNetV2, with advanced fire detection techniques. The incorporation of the SBiLSTM-AM model allows for enhanced sequential data processing. Additionally, implementing the SHO fine-tunes the model, improving the accuracy and efficiency of fire detection in complex environments.

## Literature survey

Kumar et al.^11^ present an innovative method incorporating face detection technology and navigation abilities. Utilizing the power of the Raspberry Pi single-board computer, this method offers an efficient solution and is cost-effective. The face recognition part of this method employs computer vision and advanced ML models. The navigation feature of the technique utilizes a combination of fire detection, obstacle detection, panic detection, and face detection using a Raspberry Pi camera module and fire sensor button. Singh et al.^12^ projected the progressive blind stick by incorporating an obstacle detector in front of the consumer, a fire detector in the neighbouring environment, and IoT incorporation for real-world information conveyed to the website. In^13^, the design of a model focused on supporting those who are visually impaired or blind people. Blind people might attain individual mobility using a blind stick that helps them navigate. Persons with vision impairments often depend on external assistants, like computer devices, trained canines, or human beings. This paper attained this target by involving ultrasonic and buzzer sensors to help the consumer overcome these difficulties. The recommended method will utilize ultrasonic sensors to offer accurate guidance to the consumer on element position. In^14^, the smart blind stick defined in this research was designed to assist VIPs in maintaining their safety and mobility. The smart blind stick is a reasonably priced and cutting-edge gadget that associates many communication modules and sensors to help users possibly spot risks and avoid them. Tesfaye^15^ aimed to implement and design electronic travel support for visually impaired pedestrians, employing a GSM module, infrared technology, and ultrasonic sensors. The smart cane incorporates IR technology and ultrasonic sensors to identify obstacles utilizing IR signals and ultrasonic waves. In addition, this method is furnished with a GSM module, enabling it to send SMS notifications to selected contacts in case of emergencies. Pressing an emergency button sends a message to a particular phone number. Oureshi et al.^16^ apply smart devices to make daily activities easier for each category of blind individuals. These smart devices can use picture processing and AI to identify diverse objects, colours, and faces. Abuelmakarem et al.^17^ project an intelligent cane for VIB individuals incorporated with IoT. These smart gadgets promote confidence and independence for VIB people.

In^18^, the concept of the smart stick intends to offer smart electronic support to the visually impaired. The method utilizes the Voice Module and Arduino UNO to deliver real-world assistance and artificial vision. This paper mainly targets the visually impaired, who cannot move around individually. This method contains ultrasonic sensors, and feedback is received by voice. The system-wide objective is to offer visually impaired individuals by delivering data about scenarios around dynamic and static objects in their surroundings. Xie et al.^19^ propose an AIoT-integrated Digital Twin for real-time fire monitoring and forecasting in multi-floor buildings, using the AutoDecoder Long Short-term Memory Neural Network (ADLSTM-Fire) model to predict fire development. Tejani et al.^20^ present the 2-archive Multi-Objective Cuckoo Search (MOCS2arc) approach, which improves optimization by minimizing mass and compliance in truss structures and ZDT test functions. Dzeng, Fan, and Tian-Lin^21^ developed a dynamic-threshold collision alert system for construction equipment, improving accuracy by considering object types, orientation, and distances while reducing false alarms. Nonut et al.^22^ propose a metaheuristic-based method for system identification of small-scale fixed-wing UAVs, dividing the aerodynamic model into longitudinal and lateral dynamics. Aye et al.^23^ introduce a multi-fidelity, multi-objective surrogate-assisted optimization for airfoil shape, maximizing lift-to-drag ratio with geometry constraints. It utilizes Computational Fluid Dynamic (CFD) and XFoil for high and low-fidelity simulations and improves the surrogate model with infill sampling. Duggi, Rafiei, and Salehi^24^ present benchmark datasets for three application types: DNN inference, ML inference, and video transcoding across diverse clouds. Cai et al.^25^ investigate the effect of nitrogen injection into a closed tunnel on fire-fighting effectiveness, concentrating on the impact on oxygen concentration, combustion, and smoke production. He et al.^26^ present a real-time, scale-adaptive visual tracking method based on Best Buddies Similarity (BBS), designed to handle nonrigid deformation and perspective changes in bionic robots for improved environmental perception and movement control. Kiamansouri^27^ emphasizes the critical role of programming in advancing renewable energy solutions, focusing on optimizing energy systems, enabling smart grids, and driving the transition to sustainable energy in Iran and globally.

Sun et al.^28^ review the Underwater Vehicle-Manipulator System’s (UVMS) development, examining its challenges, limitations, and future directions. Sun et al.^29^ propose a service function chain (SFC) deployment optimization algorithm using breadth-first search (BFS) to find the shortest path and prioritize paths with fewer hops for deployment. Performance is compared with the greedy and simulated annealing (G-SA) algorithms. Cui et al.^30^ present an autonomous navigation framework for intelligent wheelchairs, utilizing multi-sensor integration and hierarchical cost maps for precise path planning, obstacle avoidance, and real-time safety in dynamic outdoor environments. Wang et al.^31^ present a robotic teleoperation system integrating augmented reality (AR) and robotic arm operations to enable telekinetic control. Zhao et al.^32^ introduce Causal Intervention Visual Navigation (CIVN), incorporating deep reinforcement learning (DRL) with causal intervention through Causal Attention. This approach enhances visual navigation by addressing confounding effects, enhancing representation quality and reducing navigation issues. Qiao et al.^33^ examine the tourism experiences of wheelchair users, using embodiment theory to explore their experiences across three stages: body appearance, presence, and departure, and provide recommendations for enhancing accessibility for disabled tourists. He et al.^34^ introduce a Semantic-Group Textual Learning (SGTL) and Vision-guided Knowledge Transfer (VGKT) module, integrating semantic-based text grouping with vision-guided attention for extracting relevant textual features. The relational knowledge transfer adapts visual cues to improve textual representation, enhancing the alignment between vision and language. Fan, Lei, and Yang^35^ present a one-stage object detection framework using mixed data augmentation, a novel backbone enhancement strategy, and shape-aware loss to enhance detection accuracy, particularly for small and irregularly shaped targets. Zheng et al.^36^ studied the relationship between coverage criteria and DNN fairness across diverse models and datasets. Gu et al.^37^ introduce the Siamese Manhattan LSTM-SNP (SiMaLSTM-SNP) approach for sentence recordering (SR), integrating Word2vec, 10-layer Attention, and a Siamese LSTM-SNP structure. It utilizes multi-head self-attention to capture text associations and calculates the relatedness score with Manhattan distance. Ding et al.^38^ introduce dialogue emotion recognition (DialogueINAB), a neural network model for emotion recognition in conversations, utilizing attitude behaviour theory. It utilizes crossmodal transformers to simulate the interaction of interlocutors’ attitudes and speech behaviour for emotion detection.

The limitations of the existing studies comprise the reliance on conventional object detection and navigation methods, which may face difficulty with dynamic, complex environments, especially in scenarios with self-obstruction or varying object movements. While several approaches focus on specific domains like fire detection, blind assistance, or wheelchair navigation, there is limited integration across these domains, affecting the development of a more holistic solution. Furthermore, although advanced sensors and ML methods have improved accuracy, the models often encounter real-time processing and scalability challenges, specifically in unpredictable real-world conditions. Additionally, fairness in DNN models remains a significant challenge, with limited exploration of the correlation between coverage criteria and fairness metrics. There is also a gap in applying causal interventions to address confounding effects in tasks like visual navigation, and the integration of knowledge transfer techniques to improve model performance is underexplored. Lastly, incorporating renewable energy systems is critical, but integrating smart grids and system optimization is still in the early stages, needing additional research to scale effectually.

## The proposed methodology

**Fig. 1:**
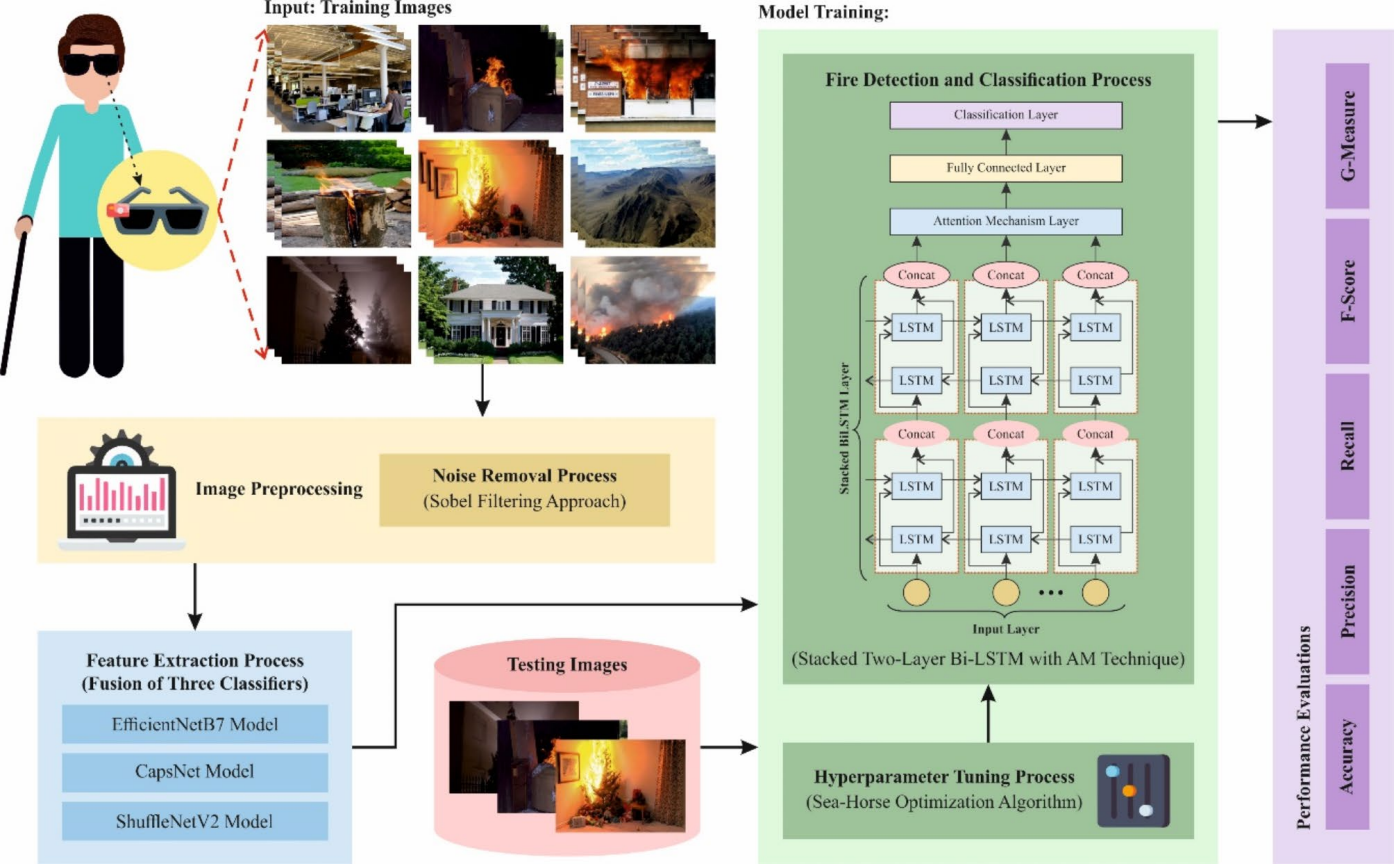
Overall process of SFDAB-ARNNSHO model.

This manuscript presents a novel SFDAB-ARNNSHO methodology. The main intention of the SFDAB-ARNNSHO methodology is to detect and classify fire for blind people. It encompasses four major steps: image pre-processing, a fusion of feature extractors, fire detection using SBiLSTM-AM, and an SHO-based parameter optimizer process. Figure 1 defines the entire procedure of the SFDAB-ARNNSHO model.

### Stage I: image pre-processing

Initially, the proposed SFDAB-ARNNSHO model performs the image pre-processing by utilizing SF to remove noise in input data^39^. This model is chosen due to its simplicity and efficiency in edge detection. It emphasizes areas of rapid intensity change, which helps detect the boundaries of objects like fire, which often have sharp contrasts. Unlike more complex methods, SF is computationally effectual and can be easily integrated into real-time systems, making it ideal for applications like fire detection. Its capability to highlight edges assists in mitigating the noise and improves the visibility of crucial features, resulting in improved detection accuracy. Furthermore, SF is less prone to overfitting due to more complex filters, ensuring stable and reliable performance in diverse environments. These advantages make it an appropriate choice over other techniques, such as Gaussian or Laplacian filters, which may not be as effective in edge localization or computationally efficient.

SF is an influential image processing model employed to perceive edges by emphasizing regions of major strength variations in an image. Smart fire recognition methods mainly classify fire-related features like smoke edges, flame contours, or hotspots. The technique removes vital data about latent fire hazards by employing SF to picture or thermal camera inputs. This filtered data improves the accuracy of recognition approaches, decreasing false positives and enhancing response times. To help the blind, the system interprets the processed data into available alerts, like vibrations or audio signals, certifying appropriate warnings. This combination of SF with adaptive outputs makes the system trustworthy and complete for visually challenged individuals.

### Stage II: fusion of feature extraction

Furthermore, the fusion of the feature extractor adopts three methods EfficientNetB7, CapsNet, and ShuffleNetV2. This hybrid model provides a robust incorporation for feature extraction due to their complementary strengths. EfficientNetB7, known for its high accuracy and efficiency, utilizes a scaling method that optimizes the network’s depth and width, making it highly effective for complex feature extraction in massive datasets. CapsNet, with its capsule network architecture, outperforms in recognizing spatial hierarchies and preserving the relationship between parts of an object, which improves the robustness of the model to discrepancies in input data. ShuffleNetV2, designed for lightweight mobile applications, balances computational efficiency and high performance, making it ideal for real-time fire detection. Integrating these three methods benefits the model from diverse features, ensuring more comprehensive and robust feature learning related to a single process. This fusion allows the model to handle diverse challenges such as scale variation, rotation, and noise, making it more adaptable and accurate in detecting fire in diverse environments.

#### EfficientNetB7 classifier

Owing to the higher improvement in the network depth and width, convolution layers, deep CNN (DCNN) structures are typically overloaded, making a structure computationally costly and cooperating with system efficiency^40^. There are exchanges between network efficiency and accuracy. The authors presented a family of EfficientNet sequences, namely EfficientNetB0-B7, as a backbone architecture that keeps outshining numerous DCNN-based architectures, like Inception-V2, ResNet, Inception-V3, $\:ResNet50$, and DenseNet. This challenges standard scaling methods utilized by previous studies, which randomly improve the system’s width, resolution, and depth to enhance the generalizability. Compound scaling emerged from balancing the system dimensions of depth $\:d$, resolution $\:r$, and width $\:w$, by scaling it by an infinite ratio as represented in Eq. (1).

1 $$\:d=\alpha\:{\varnothing},w=\beta\:{\varnothing},\:r=\gamma\:{\varnothing}\:$$

Such that$\:\:\alpha\:.\beta\:2.y2\approx\:2$ while $\:\alpha\:\ge\:1,\beta\:\ge\:1,y\ge\:1.\alpha\:,\beta\:$ and $\:y$ values are described by the grid searching method. A user’s definable parameters describe the increase in computing sources to the system is identified as $\:{\varnothing}$. Flops of the convolutional networking process are equal to $\:w2$, $\:r2,\:$and $\:d$. Fops should be doubled when the network depth is doubled. Simultaneously, fops should be improved 4 times when the width and resolution are doubled. The rise in fops is depending the relation $\:(\alpha\:.\beta\:2.y2){\varnothing}$, so complete fops are detailed by $\:2{\varnothing}$ for new values. The EfficientNet architecture contains stem blocks, which follow the 7 blocks and final layers. All blocks in EfficientNet comprise the different module counts, and the number of modules rises as it benefits from EfficientNet-B0-B7. It comprises variable depth and parameters. EffcientNetB0 is the simplest version of EfficientNet using 5. 237 layers and$\:\:3M$ parameters, while EffcientNet-B7 contains 66 M and 813 layers. EfficientNet architecture uses MB $\:Conv$ layers related to MnasNet and MobileNet-V2. In the meantime, the layer of normalization is pre-existing within the stem layer. Thus, no other image normalization is required as a pre-processing phase, and thus, it captures an input image inside the 0255 range. Five differences of pretrained EfficientNet, namely EfficientNet B0‐B4, maintain the classification. The disorders for selecting EfficientNet difference are according to dissimilar variables: dataset size, the resource available for the network parameter, assessment and model training, model depth, and batch size.

#### CapsNet model

The CapsNet model contains four main parts: the initial caps, the standard convolutional, the input, and the output caps layers^41^. The resultant capsule counts in the resultant caps layer are equivalent to the sum of condition categories, and the length of every resultant capsule characterizes the occurrence likelihood of some condition category. The condition category equivalent to the greatest likelihood of occurrence is the last identified condition category.

This input layer transforms the real-time raw acceleration signals into 2-D images; the raw signals fulfil the image pixels in sequence. Based on guaranteeing the continuance of the raw signal, a 2D association of nonadjacent data is advantageous for the system to remove the signal association in the nonadjacent interval. The standard convolutional layer has been utilized to detect local combinations of characteristics in the unique input and to map their arrival for mapping the feature. The main caps layer represents the convolutional capsules applied to distribute the progressive look of signal patterns into low-level initial capsules. During this output layer, the dynamic routing model ensured that the initial capsules moved their data to the maximum related resultant capsule.

The CapsNet loss is stated as Eq. (2), while the loss of margin $\:Los{s}_{k}$ for every output capsule:

2 $$\:Los{s}_{all}={\sum\:}_{k}Los{s}_{k}$$

3 $$\:Los{s}_{k}={C}_{k}\text{m}\text{a}\text{x}(0,{m}^{+}-\Vert\:{\nu\:}_{k}\Vert\:{)}^{2}+{\lambda\:}_{1}\left(1-{c}_{k}\right)\text{m}\text{a}\text{x}(0,\:\Vert\:{\nu\:}_{k}\Vert\:-{m}^{-}{)}^{2}$$

while $\:\Vert\:{\nu\:}_{k}\Vert\:$ signifies the complete length of the output capsule vector, if condition category $\:k$ exists, $\:{C}_{k}$ is equivalent to 1, representing that simply the initial word of the function of loss is calculated for the consistent $\:k\:th$ capsule. Still, for another capsule, simply the next word is calculated. Moreover, $\:{m}^{+}$ and $\:{m}^{-}$ are fixed at 0.9 and 0.1. This means if $\:{C}_{k}=1$, the loss for the $\:k\:th$ capsule of output should be $\:\text{z}\text{e}\text{r}\text{o}$ when forecasting the accurate condition category $\:k$ using a likelihood better than 0.9, and it should be $\:non-zero\:$when the likelihood is lower than 0.9. Likewise, if $\:{C}_{k}=0$, the loss should be $\:0$ when the capsule forecasts the improper condition category by likelihood lower than 0.1 and $\:non-zero\:$when the possibility is greater than 0.1. The down-weighted parameter $\:{\lambda\:}_{1}$ is fixed to 0.5 as default.

#### ShuffleNetV2

**Fig. 2:**
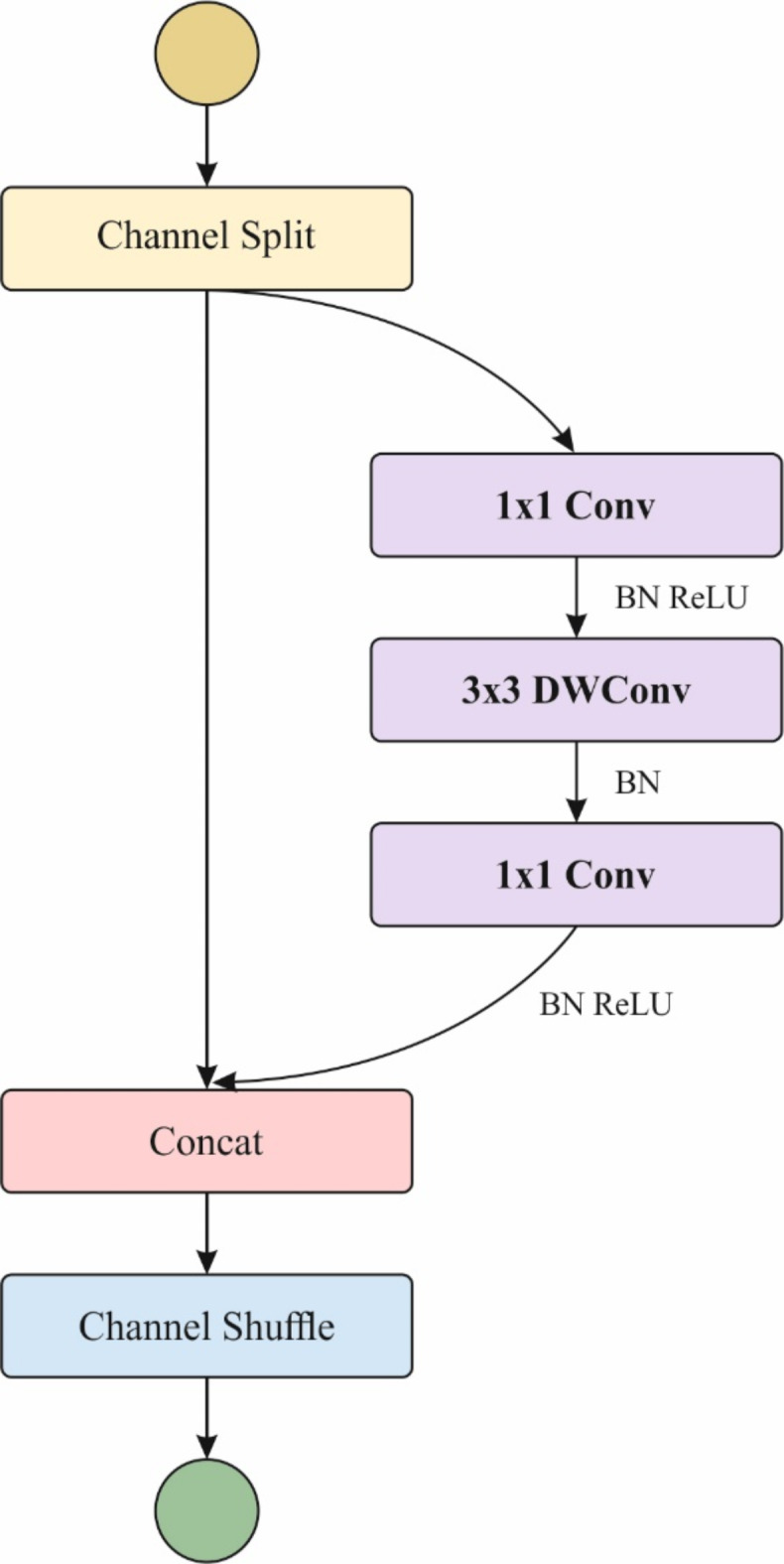
Architecture of ShuffleNetV2.

ShuffleNetV1 is an effective and lightweight CNN method designed for embedded devices^42^. This system considerably decreases the model parameter counts while preserving great precision over methods like grouped convolutions, channel shuffle, and depthwise separable convolutions. Building on the general mechanisms of ShuffleNetV1, ShuffleNetV2 is intended according to the following four principles: (i) keeping constant input and output channel dimensions in convolution layers for maximizing the speed of the model; (ii) carefully utilizing grouped convolutions to prevent improving memory access costs; (iii) decreasing the number of model branches to improve speed; and (iv) reducing tensor processes to drop time consumption. Figure 2 depicts the structure of ShuffleNetV2.

ShuffleNetV2 is made of elementary units and down-sampling units. During the elementary unit, the input characteristics are divided into dual branches: the left branch permits directly over network layers, whereas the right branch implements a sequence of $\:1\text{x}1$ convolutional, $\:3\text{x}3$ depth-wise convolution, and $\:1\times\:1$ convolution, accompanied by channel connection. The right branch carried out $\:1\text{x}1$ convolution, $\:3\text{x}3$ depth-wise convolutional using a stride of 2, and other $\:1\text{x}1$ convolutions to decrease spatial dimensions and remove features. The left branch carried out $\:3\text{x}3$ depth-wise convolutions by a stride of 2, succeeded by $\:1\text{x}1$ convolutions. The outputs of these dual branches are then connected through channel shuffle, allowing effective feature extraction but preservative information integrity and meaningfully enhancing model computational complexity. However, ShuffleNet-V2-1.0 outshines computational cost; it faces challenges like exchanges amongst computational complexity and model dimensions, insufficient multitask adaptability, limited feature expression, and complexity in tuning and training. Additional investigation and developments are required to improve the performance of the models.

### Stage III: fire detection using SBiLSTM-AM

**Fig. 3:**
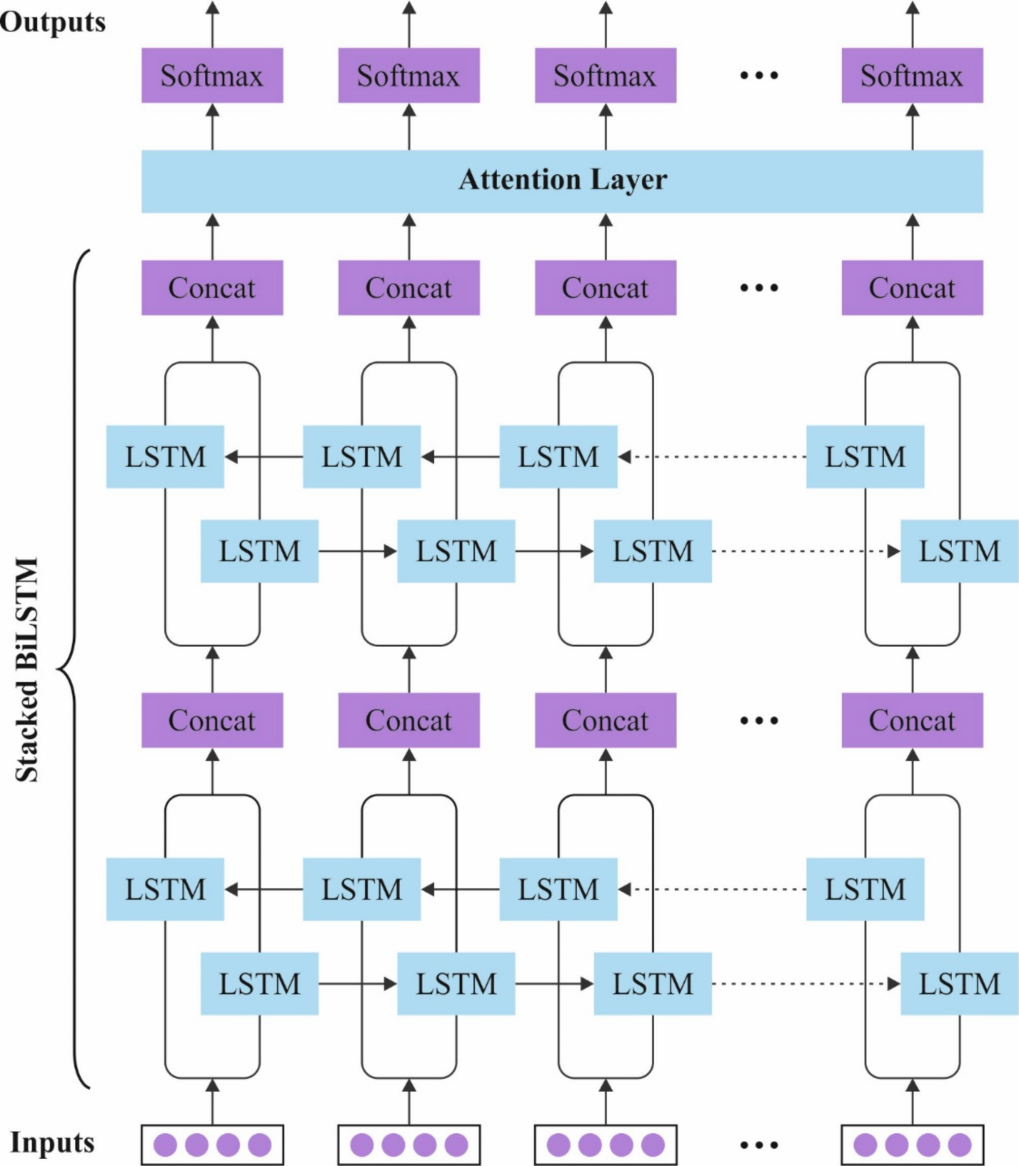
Architecture of SBiLSTM-AM model.

In addition, the SFDAB-ARNNSHO model performs fire detection and classification using the SBiLSTM-AM technique^43^. This method is chosen because it captures spatial and temporal dependencies in sequential data. It is significant for comprehending fire patterns in video or time-series data. The bidirectional LSTM allows the model to process information in both forward and backward directions, capturing context from past and future frames and improving its ability to detect fires accurately over time. The attention mechanism refines the model by concentrating on the most relevant features, enhancing classification performance and mitigating false positives. This is specifically valuable in fire detection, where quick and accurate identification of critical features is crucial. Additionally, SBiLSTM-AM effectively handles variable-length sequences and noise, making it more resilient to conventional methods like CNNs that may face difficulty with sequential dependencies. By incorporating context awareness and feature attention, SBiLSTM-AM gives a robust approach to fire detection, outperforming simpler models in handling dynamic and complex fire events. Figure 3 illustrates the structure of the SBiLSTM-AM method.

LSTM is a unique variation of RNN, which is obtained to tackle the task of trapping longer-term dependencies and relieving problems such as exploding or vanishing gradients combatted by CNN. The developed method covers dual layers stacked BiLSTM to take forward and backward dependencies. The input sequence like $\:{x}_{1},{x}_{2},{x}_{3}\cdots\:{x}_{f}$ arrives the BiLSTM initial hidden layer (HL) for $\:the\:tth$ time. It moves over dual directions like the forward direction $\:({a}_{1},{x}_{2},{a}_{3}\dots\:{a}_{t}$), which is employed to collect contextual data from every preceding time step. In contrast, the backward direction $\:({c}_{1},{c}_{2},{c}_{3}\cdots\:{c}_{f}$) collects data from every incoming time step. Lastly, every output layer is combined and served to an attention layer. Every LSTM cell contains memory cell $\:{U}_{t}$ with 3 gates termed input gate $\:{i}_{t},$ an output gate $\:{o}_{t}$ and forget gate $\:{f}_{t}$. The relation among the distinct gates, HL, and memory cells is explained below for the early forward layer and HL.

4 $$\:{i}_{t}^{a}=\sigma\:\left({U}_{i}^{a}{x}_{t}+{W}_{i}^{a}\text{a}\left(t-1\right)+{b}_{i}^{a}\right)$$

5 $$\:{f}_{t}^{a}=\sigma\:\left({U}_{f}^{a}{x}_{t}+{W}_{f}^{a}a\left(t-1\right)+{b}_{f}^{a}\right)$$

6 $$\:{o}_{t}^{a}=\sigma\:\left({U}_{o}^{a}{x}_{t}+{W}_{0}^{a}a\left(t-1\right)+{b}_{o}^{a}\right)$$

7 $$\:{U}_{t}^{a}=\text{t}\text{a}\text{n}\text{h}\left({U}_{u}^{a}{x}_{t}+{W}_{u}^{a}a\left(t-1\right)+{b}_{u}^{a}\right)$$

8 $$\:{C}_{t}^{a}=({i}_{t}^{a}*{U}_{t}^{a}*{f}_{t}^{a}C\left(t-1{)}^{a}\right)$$

9 $$\:{a}_{t}={o}_{t}^{a}*tanh{C}_{t}^{a}$$

The below-mentioned formulations are employed to calculate the HL $\:{b}_{t}$ for the 2nd forward layer:

10 $$\:{i}_{t}^{b}=\sigma\:\left({U}_{i}^{b}{x}_{t}+{W}_{i}^{b}b\left(t-1\right)+{b}_{i}^{b}\right)$$

11 $$\:{f}_{t}^{b}=\sigma\:\left({U}_{f}^{b}{x}_{t}+{W}_{f}^{b}b\left(t-1\right)+{b}_{f}^{b}\right)$$

12 $$\:{o}_{t}^{b}=\left({U}_{o}^{b}{x}_{t}+{W}_{0}^{b}b\left(t-1\right)+{b}_{o}^{b}\right)$$

13 $$\:{U}_{t}^{b}=\text{t}\text{a}\text{n}\text{h}\left({U}_{u}^{b}{x}_{t}+{W}_{u}^{b}b\left(t-1\right)+{b}_{u}^{b}\right)$$

14 $$\:{C}_{t}^{b}=({i}_{t}^{b}*{U}_{t}^{b}*{f}_{t}^{b}C\left(t-1{)}^{b}\right)$$

15 $$\:{b}_{t}={o}_{t}^{b}*\text{t}\text{a}\text{n}\text{h}{C}_{t}^{b}$$

The prescribed relations for 1st backward layer and the HL $\:{c}_{t}$ are given as follows:

16 $$\:{i}_{t}^{c}=\sigma\:\left({U}_{i}^{c}{x}_{t}+{W}_{i}^{c}c\left(t-1\right)+{b}_{i}^{c}\right)$$

17 $$\:{f}_{t}^{c}=\sigma\:\left({U}_{f}^{c}{x}_{t}+{W}_{f}^{c}c\left(t-1\right)+{b}_{f}^{c}\right)$$

18 $$\:{o}_{t}^{c}=\sigma\:\left({U}_{o}^{c}{x}_{t}+{W}_{0}^{c}c\left(t-1\right)+{b}_{o}^{c}\right)$$

19 $$\:{U}_{t}^{c}=\:\text{t}\text{a}\text{n}\text{h}\:\left({U}_{u}^{c}{x}_{t}+{W}_{u}^{c}c\left(t-1\right)+{b}_{u}^{c}\right)$$

20 $$\:{C}_{t}^{c}=({i}_{t}^{c}*{U}_{t}^{c}*{f}_{t}^{c}C\left(t-1{)}^{C}\right)$$

21 $$\:{c}_{t}={o}_{t}^{c}*\text{t}\text{a}\text{n}\text{h}{C}_{t}^{c}$$

The correct relations for the 2nd backward layer and the HL $\:{d}_{t}$ are below.

22 $$\:{i}_{t}^{d}=\sigma\:\left({U}_{i}^{d}{x}_{t}+{W}_{i}^{d}d\left(t-1\right)+{b}_{i}^{d}\right)$$

23 $$\:{f}_{t}^{d}=\sigma\:\left({U}_{f}^{d}{x}_{t}+{W}_{f}^{d}d\left(t-1\right)+{b}_{f}^{d}\right)$$

24 $$\:{o}_{t}^{a}=\sigma\:\left({U}_{o}^{a}{x}_{t}+{W}_{0}^{a}d\left(t-1\right)+{b}_{o}^{d}\right)$$

25 $$\:{U}_{t}^{d}=\:\text{t}\text{a}\text{n}\text{h}\:\left({U}_{u}^{d}{x}_{t}+{W}_{u}^{d}d\left(t-1\right)+{b}_{u}^{d}\right)$$

26 $$\:{C}_{t}^{d}=({i}_{t}^{d}*{U}_{t}^{d}*{f}_{t}^{d}C\left(t-1{)}^{d}\right)$$

27 $$\:{d}_{t}={o}_{t}^{d}*\text{t}\text{a}\text{n}\text{h}{C}_{t}^{d}$$

In the present work, attention models are recommended to avoid a few analyses performed by pooling operators. An encoder uses numerous weights and values for the phrase’s words, interpreting the HLs of an entire sentence into a vector model.

28 $$\:{\alpha\:}_{t}=\frac{exp\left(VT.{h}^{-}\right)}{{\sum\:}_{t}exp\left(V.{h}^{-}\right)}\:$$

29 $$\:{S}_{Aw}={\sum\:}_{t}{\alpha\:}_{t}{h}_{t}$$

Here, $\:h$ and $\:{h}^{-}$ denote the forward and backward HLs from Bi-LSTM, and $\:V$ denotes a trainable parameter. $\:{S}_{Aw}$ represents an average of transferred weights to ISTM HLs.

30 $$\:\overrightarrow{{h}_{tLSTM}}=\overrightarrow{\left(LSTM\right)}\left({c}_{t}\right),t\in\:\left(1,\:m\right)\:$$

31 $$\:\overleftarrow{{h}_{tLSTM}}=\overleftarrow{\left(LSTM\right)}\left({c}_{t}\right),t\in\:\left(m,\:1\right)$$

An explanation for every word, $\:{W}_{t}$, is obtained by concatenating forward and backward directions in the formulation below.

32 $$\:{h}_{tLSTM}=\overrightarrow{{h}_{tLSTM}}\oplus\:\overleftarrow{{h}_{tLSTM}}\:$$

The attention technique was employed for the $\:{h}_{tLSTM}$ technique, which allows it to give less or more attention to several terms in the commentary. On $\:{h}_{tLSTM}$, the attention model allows the model to yield specific words in the sentence with less or more attention. To achieve this, the vector of features is upgraded by isolating useful terms in the comment, as presented in Eq. (33).

33 $$\:{u}_{tLSTM}=\text{t}\text{a}\text{n}\text{h}\left(\left({W}_{wLSTM}\cdot\:{h}_{tLSTM}\right)+{b}_{wLSTM}\right)$$

34 $$\:{\alpha\:}_{tLSTM}=\frac{exp\left({u}_{t}^{{T}_{LSTM}}\right)\cdot\:{u}_{wLSTM}}{{\sum\:}_{t}exp\left({u}_{t}^{{T}_{LSTM}}\cdot\:{u}_{wLSTM}\right)}$$

35 $$\:{S}_{LSTM}={\sum\:}_{t}({\alpha\:}_{tLSTM}\cdot\:{h}_{tLSTM})$$

### Stage IV: SHO-based parameter optimizer

**Fig. 4:**
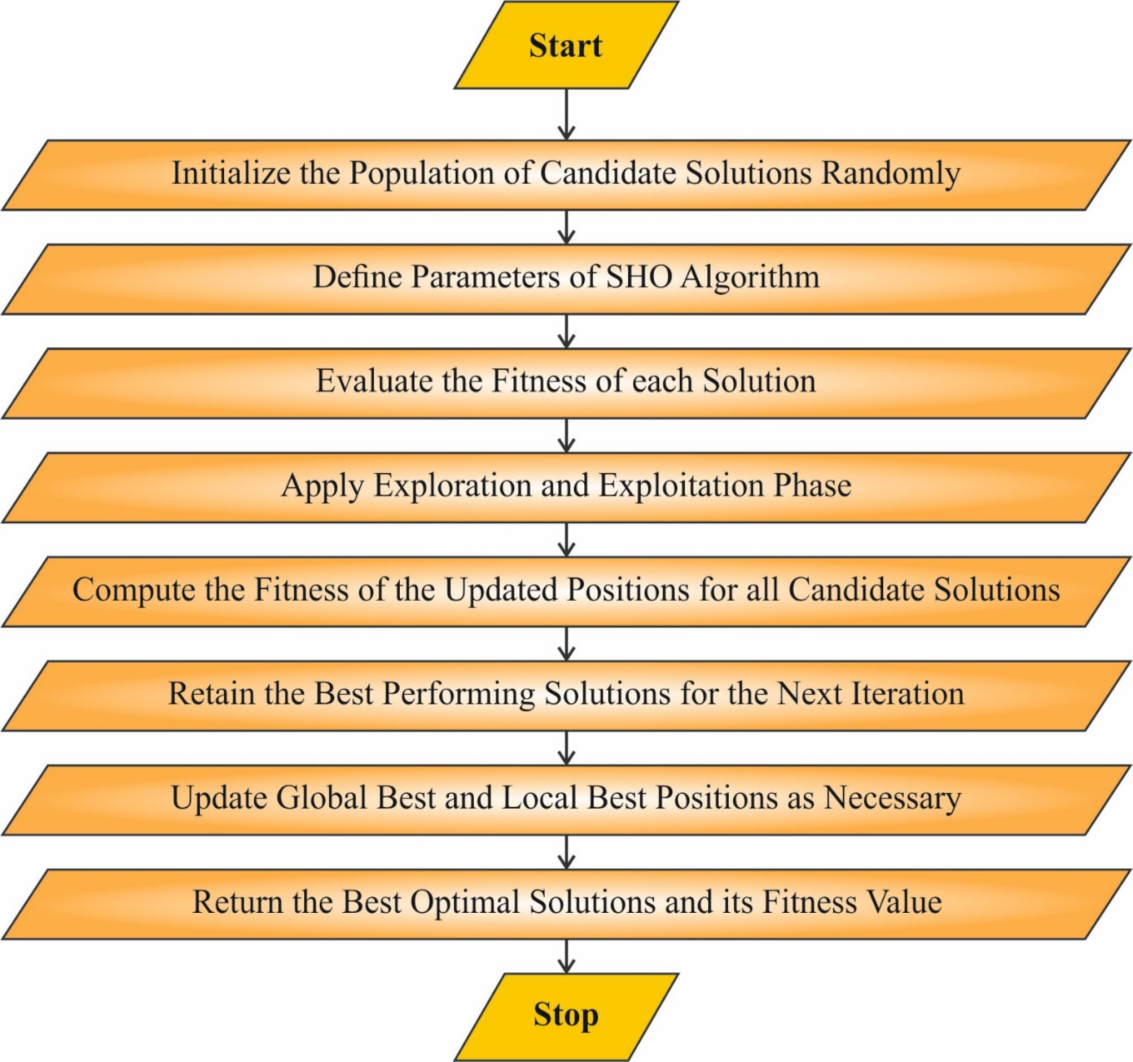
Steps involved in the SHO method.

Eventually, the hyperparameter tuning of the SBiLSTM-AM method is accomplished by implementing the SHO model^44^. This methodology is chosen for its ability to effectively fine-tune hyperparameters and improve the performance of the fire detection model. SHO is motivated by natural phenomena, making it a unique and powerful global optimization technique that avoids local minima, a common issue with other methods like gradient descent. It is specifically beneficial for optimizing complex, multi-dimensional spaces with non-linearities, which is often the case in DL models. Unlike conventional optimization algorithms, SHO adapts dynamically, ensuring improved convergence to optimal solutions with fewer iterations. Its capability to balance exploration and exploitation during the optimization process allows it to find better-performing configurations without exhaustive search, enhancing the overall efficiency and accuracy of the model. This results in superior fire detection capabilities compared to conventional techniques, such as grid or random search, which may require more time and computational resources to achieve similar results. Figure 4 specifies the structure of the SHO methodology.

The SHO is presented to pretend a variation of the sea horse’s behaviours in the ocean, like Brownian and spiral motions. Consistent behaviours should influence the sea horse’s behaviour to update its location.

## Population initialization

In SHO, initial populations are generated at random in the search space. Assume that all sea horses characterize a solution candidate within the problem-searching space; the mathematic equation to initialize a sea horse is as shown:

36 $$Seahorses~=\left[ {\begin{array}{*{20}{l}} {x_{1}^{1}}& \cdots &{x_{1}^{{Dim}}} \\ \vdots & \ddots & \vdots \\ {x_{{pop}}^{1}}& \cdots &{x_{{pop}}^{{Dim}}} \end{array}} \right]$$

whereas the formulation for the $\:i\:th$ individual is:

37 $$\:{X}_{i}=\left[{x}_{i}^{1},\:\dots\:,\:{x}_{i}^{Dim}\right]$$

38 $$\:{x}_{i}^{j}=rand\:\times\:\left(U{b}^{j}-L{b}^{j}\right)+L{b}^{j}$$

$\:Ub$ and $\:Lb$ represent upper and lower limits; $\:rand$ signifies the randomly generated number amongst 0 and 1, $\:and\:i$ and $\:j$ signify the positive integer amongst [1, pop] and$\:\:[1,\:Dim].$

### Seahorses motion behaviour

This sea horse utilizes Brownian and spiral motion to regenerate the location. The spiral motion is mainly applied in the local exploitation stage after the seahorse mimics Lévy flight to measure the length of the movement step. To balance the exploration and exploitation of the model, $\:{r}_{1}=0$ is employed as the boundary division, and it should be utilized $\:{r}_{1}>0$ for local exploitation directly and $\:{r}_{1}\le\:0$ for global exploration.

1. **Spiral motion**.

Once the normal randomly generated number is $\:{r}_{1}>0$, the sea horse will address the elite individual $\:{X}_{elite}$ based on the spiral motion, which is concentrated on the local exploitation phase. Lévy flight has been applied to pretend the size of the movement step after the sea horse advances in spiral motion such that the crossover location is performed with higher probability in the initial iteration to prevent extreme local exploitation.

39 $$\:{X}_{new}^{1}\left(t+1\right)={X}_{\iota\:}\left(t\right)+Levy\cdot\:(\left({X}_{elite}\:\left(t\right)-{X}_{i}\left(t\right)\right)\times\:x\times\:y\times\:z+{X}_{elite}))$$

40 $$\:x=\rho\:\times\:cos\left(\theta\:\right)$$

41 $$\:y=\rho\:\times\:sin\left(\theta\:\right)$$

42 $$\:z=\rho\:\times\:\theta\:$$

43 $$\:\rho\:=\mu\:\times\:{e}^{\theta\:\nu\:}$$

whereas $\:{X}_{elite}$ signifies the location of elite individuals, $\:x,y,$ and $\:z$ correspondingly characterize the 3D element of spiral motion, $\:\theta\:$ means the randomly generated value among $\:\left[\text{0,2}\pi\:\right]$, $\:\rho\:$ signifies stem lengths, the logarithmic spiral constant $\:\mu\:$ and $\:v$ are fixed to 0.05, the function of Levy distribution is stated as shown:

44 $$\:Levy=s\times\:\frac{\omega\:\times\:\sigma\:}{|k{|}^{\frac{1}{\lambda\:}}}$$

Here, $\:s$ denotes fixed constant 0.01, the value of $\:\omega\:$ and $\:k$ represents randomly generated numbers between zero and one, and the range of value of $\:\lambda\:$ is 0 and 2, whose formulation is demonstrated as:

45 $$\:\sigma\:=\left(\frac{\varGamma\:\left(1+\lambda\:\right)\times\:\text{sin}\left(\frac{\pi\:\lambda\:}{2}\right)}{\varGamma\:\left(\frac{1+\lambda\:}{2}\right)\times\:\lambda\:\times\:2\left(\frac{\lambda\:-1}{2}\right)}\right)$$

(2) **Brownian motion.**

Once the standard randomly generated numbers are placed $\:{r}_{1}\le\:0$, the sea horse will travel based on Brownian motion, concentrating on global exploration. It has been applied to mimic the size of the movement step to guarantee improved exploration within the searching region and prevent a fall into local ideals. The equation is as shown:

46 $$\:{X}_{new}^{1}\left(t+1\right)={X}_{i}\left(t\right)+rand\cdot\:l\cdot\:{\beta\:}_{t}\cdot\:\left({X}_{\text{i}}\right(t)-{\beta\:}_{t}\cdot\:{X}_{elite}))$$

Meanwhile, the parameter $\:l$ is fixed to a fixed value of 0.05. The arbitrary walking coefficient equation for $\:{\beta\:}_{t}$ is as demonstrated:

47 $$\:{\beta\:}_{t}=\frac{1}{\sqrt{2\pi\:}}exp\left(-\frac{{x}^{2}}{2}\right)$$

The last mathematical representation of seahorse motor behaviour is stated as follows:

48 $$\:{X}_{new}^{1}(t+1)=\left\{\begin{array}{ll}{X}_{i}\left(t\right)+Levy\cdot\:\left(\right({X}_{elite}\left(t\right)-{X}_{i}\left(t\right))\times\:x\times\:y\times\:z+{X}_{elite}(t\left)\right)&\:{r}_{1}>0\\\:{X}_{i}\left(t\right)+rand\cdot\:l\cdot\:{\beta\:}_{t}\cdot\:\left({X}_{i}\left(t\right)-{\beta\:}_{t}\cdot\:{X}_{elite}\left(t\right)\right)&\:{r}_{1}\le\:0\end{array}\right.$$

### Seahorses predation behavior

During this phase of predation behaviour, the outcome of predation is established by $\:{r}_{2}$. If $\:{r}_{2}>0.1$, the sea horse effectively chased and moved toward the food, representing the growth capability of SHO, or else representing that the predation miscarried and the sea horse sustained to discover. The location renewal equation of sea horses in predation behaviour is as demonstrated:

49 $$\:{X}_{new}^{2}\left(t+1\right)=\left\{\begin{array}{l}\alpha\:\:\left({X}_{elite}-rand\cdot\:{X}_{new}^{1}\left(t\right)\right)+\left(1-\alpha\:\right)\cdot\:{X}_{elite}\:\:\:\:\:\:if\:{r}_{2}>0.1\\\:\left(1-\alpha\:\right)\cdot\:\left(\:{X}_{new}^{1}\left(t\right)-rand.\:{X}_{elite}\right)+\alpha\:\:{X}_{new}^{1}\left(t\right)\:\:\:if{r}_{2}\le\:0.1\end{array}\right.$$

50 $$\:\alpha\:={\left(1-\frac{t}{T}\right)}^{\frac{2t}{T}}$$

Here, $\:{r}_{2}$ and $\:rand$ denote randomly generated numbers among [0,1]. $\:{X}_{new}^{1}\left(t\right)$ signifies the novel place of the seahorse after the $\:t-th$ iteration, $\:t$ and $\:T$ characterize the present iteration, and the maximal iteration count correspondingly. $\:\alpha\:$ signifies the weighting of the influencing feature.

### Seahorses breeding behaviour

Based on the fitness value level, this seahorse’s breeding behaviour separates the population into female and male groups. The best half of the optimal individuals are described as males, and the bottom half are females, which aids in obtaining the genetic features of the earlier generations.

51 $$\:{X}_{\iota\:}^{offspring}={r}_{3}\cdot\:{X}_{i}^{father}+\left(1-{r}_{3}\right)\cdot\:{X}_{\iota\:}^{mother}$$

52 $$\:fathers\:={X}_{sort}^{2}\left(1\::\frac{pop}{2}\right)$$

53 $$\:mothers\:={X}_{sort}^{2}(pop/2+1):\:pop\:)$$

Here, $\:i$ signifies a positive integer inside the interval among $\:[1,\:pop/2],$ $\:{r}_{3}$ represents randomly generated numbers amongst [0,1], $\:{X}_{sort}^{2}$ specify that $\:{X}_{new}^{2}$ is organized in sequential order of fitness value.

The SHO method has subsequent benefits. (i) Specific modifiable parameters are advantageous to the execution of the model. (ii) The exploration and exploitation of balanced models are attained by utilizing $\:{r}_{1}=0$ as the separating point between global exploration and local exploitation. (iii) The distribution pattern within the reproductive behaviour phase can get the genetic features of the earlier generation and gain improved seahorse individuals. The SHO method originates a fitness function (FF) to attain enhanced classification performance. It defines an optimistic number to signify the improved performance of the candidate outcomes. At this point, the decrease in the classifier error ratio is reflected as FF.

54 $$\begin{gathered} fitness\left( {{x_i}} \right)=ClassifierErrorRate\left( {{x_i}} \right) \hfill \\ =\frac{{Misclassified~instance~counts}}{{Total~~instance~counts}} \times 100 \hfill \\ \end{gathered}$$

## Result analysis and discussion

**Fig. 5:**
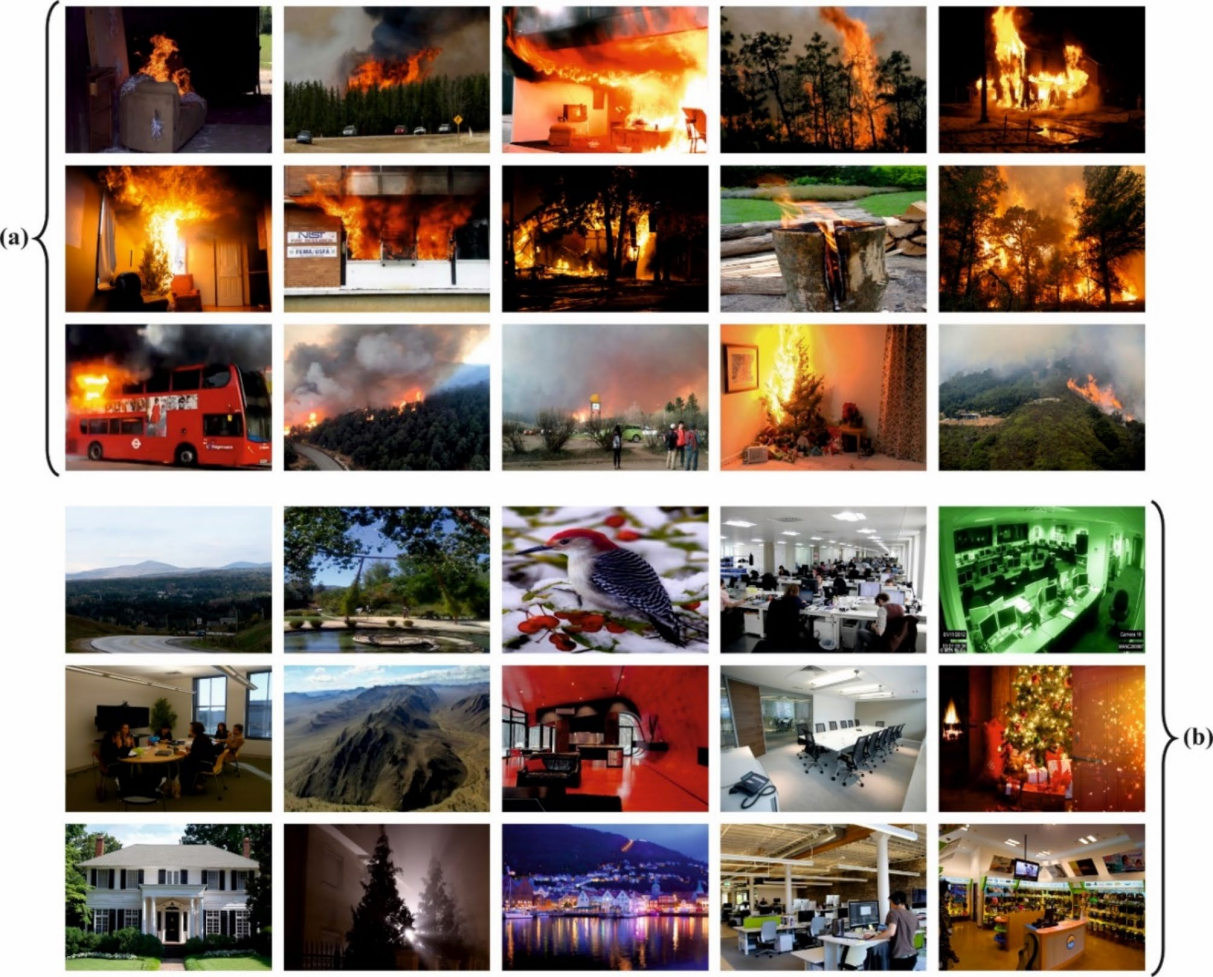
Sample of (**a**) fire and (**b**) normal.

The performance evaluation of the SFDAB-ARNNSHO technique is examined using the fire detection dataset^45^. The dataset contains 600 images in dual classes, such as fire and normal, as shown in Table 1. Figure 5 portrays a sample of fire and normal images.

**Table 1:** Details of database.

Class label	Sample images
Fire	100
Normal	500
Total images	600

**Fig. 6:**
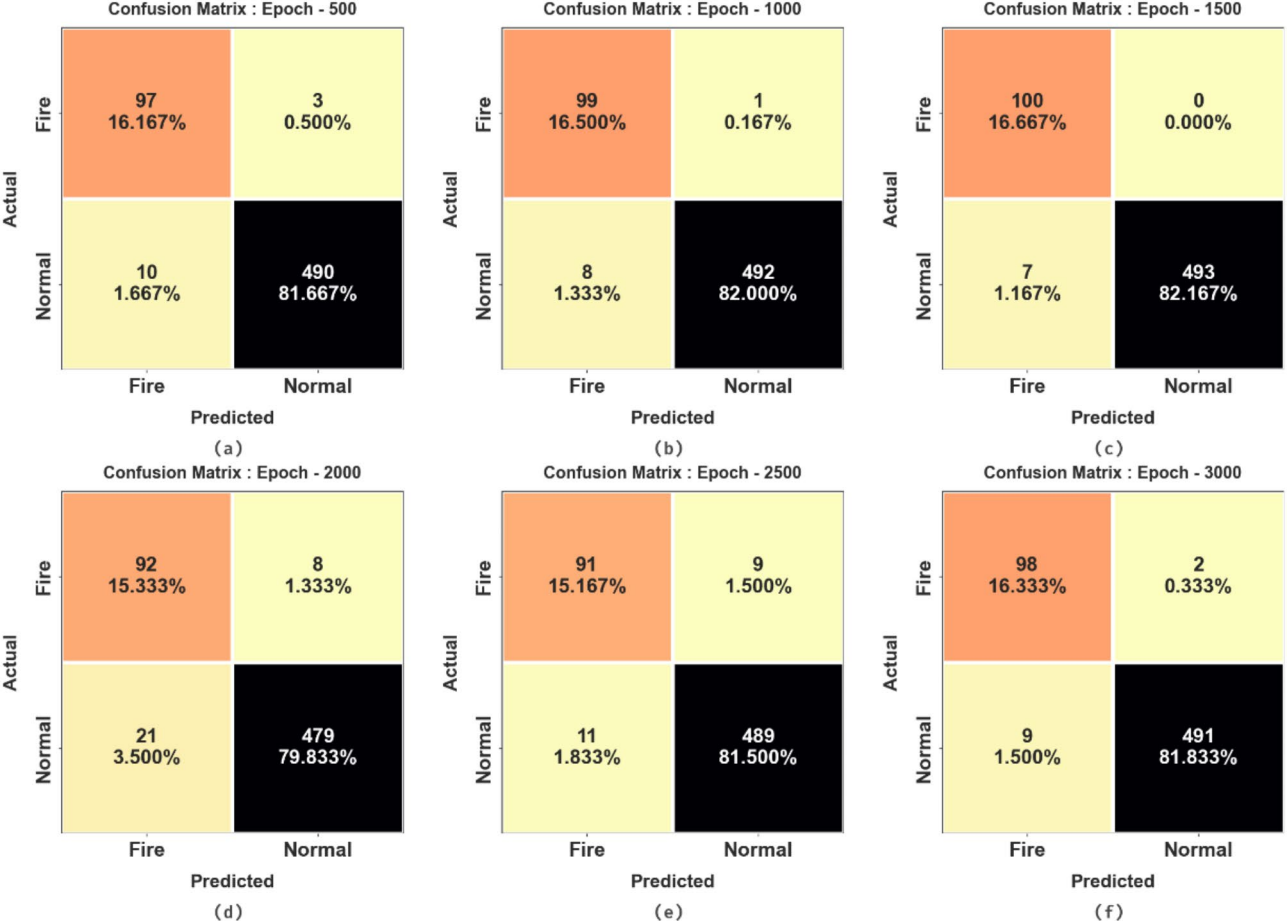
Confusion matrix of SFDAB-ARNNSHO methodology (**a**–**f**) Epochs 500–3000.

Figure 6 provides a set of confusion matrices attained by the SFDAB-ARNNSHO approach on dissimilar epochs. On 500 epochs, the SFDAB-ARNNSHO technique has identified 97 samples in the fire and 490 samples in normal. Similarly, on 1000 epochs, the SFDAB-ARNNSHO technique has identified 99 samples in the fire and 492 samples in normal. Followed by, on 1500 epochs, the SFDAB-ARNNSHO technique has identified 100 samples into the fire and 493 samples into normal. Besides, in 2000 epochs, the SFDAB-ARNNSHO technique identified 92 samples in the fire and 479 samples in normal. Finally, on 3000 epochs, the SFDAB-ARNNSHO technique has identified 98 samples in the fire and 491 samples in normal.

**Fig. 7:**
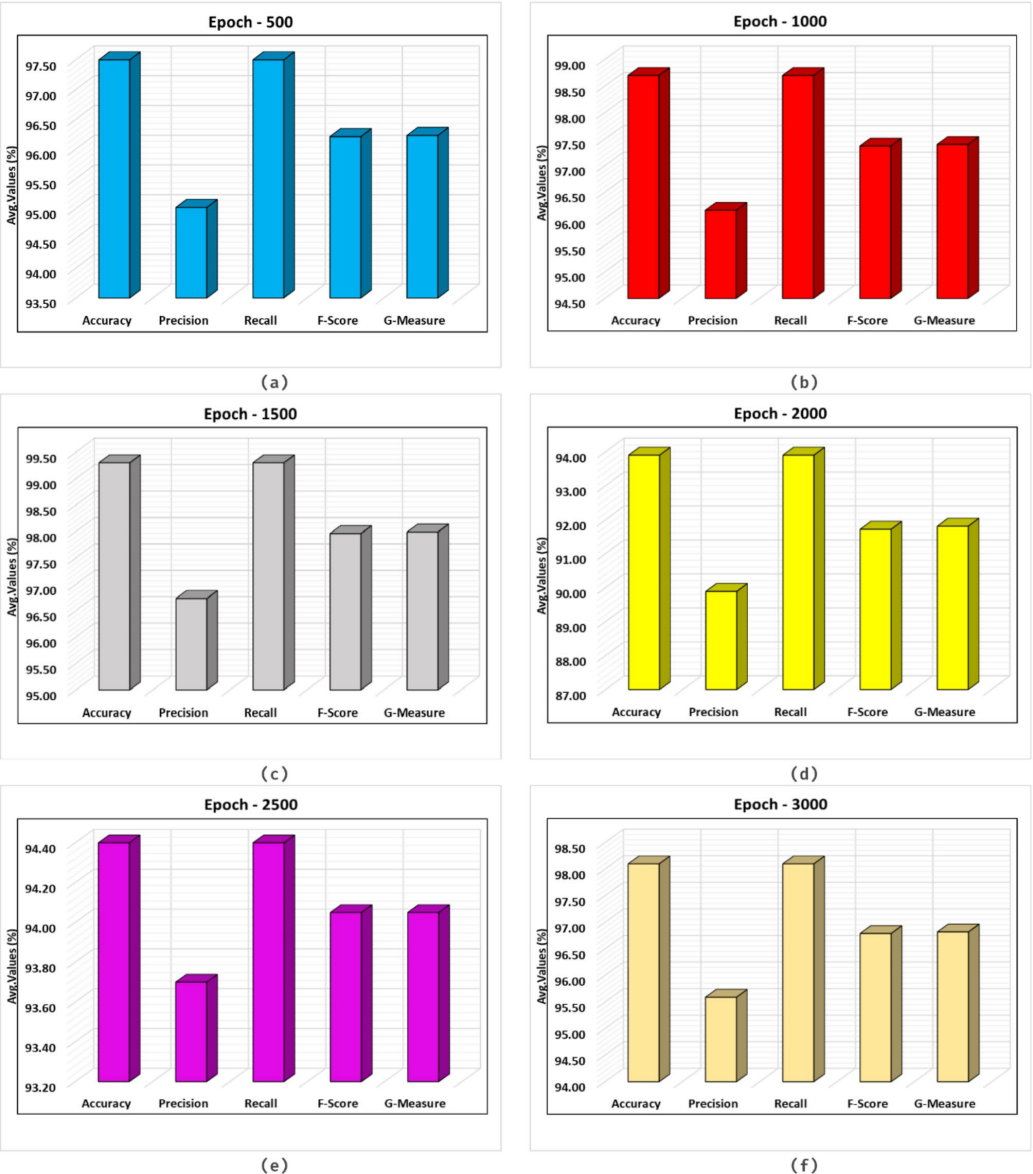
Average outcome of SFDAB-ARNNSHO approach (**a**–**f**) Epochs 500–3000.

The fire detection result of the SFDAB-ARNNSHO technique is determined under different epochs in Table 2; Fig. 7. The outcome values state that the SFDAB-ARNNSHO method correctly identified fire and standard samples. On 500 epochs, the SFDAB-ARNNSHO method provides an average $\:acc{u}_{y}$ of 97.50%, $\:pre{c}_{n}\:$of 95.02%,$\:\:rec{a}_{l}\:$of 97.50%,$\:\:\:{F}_{score}$ of 96.21%, and $\:{G}_{Measure\:}$of 96.23%. Besides, on 1000 epochs, the SFDAB-ARNNSHO method gives an average $\:acc{u}_{y}$ of 98.70%, $\:pre{c}_{n}\:$of 96.16%, $\:\:rec{a}_{l}\:$of 98.70$\:\:\:{F}_{score}$ of 97.37%, and $\:{G}_{Measure\:}$of 97.40%. Moreover, on 1500 epochs, the SFDAB-ARNNSHO method presents an average $\:acc{u}_{y}$ of 99.30%, $\:pre{c}_{n}\:$of 96.73%, $\:\:rec{a}_{l}\:$of 99.30$\:\:\:{F}_{score}$ of 97.96%, and $\:{G}_{Measure\:}$of 97.99%. At the same time, on 2000 epochs, the SFDAB-ARNNSHO approach provides an average $\:acc{u}_{y}$ of 93.90%, $\:pre{c}_{n}\:$of 89.89%, $\:\:rec{a}_{l}\:$of 93.90$\:\:\:{F}_{score}$ of 91.72%, and $\:{G}_{Measure\:}$of 91.81%. At last, based on 3000 epochs, the SFDAB-ARNNSHO approach offers an average $\:acc{u}_{y}$ of 98.10%, $\:pre{c}_{n}\:$of 95.59%, $\:\:rec{a}_{l}\:$of 98.10$\:\:\:{F}_{score}$ of 96.79%, and $\:{G}_{Measure\:}$of 96.82%.

**Table 2:** Fire detection of SFDAB-ARNNSHO methodology under dissimilar epochs.

Class labels	$Acc{u_y}$	$Pre{c_n}$	$Rec{a_l}$	${F_{score}}$	${G_{Measure}}$
Epoch − 500					
Fire	97.00	90.65	97.00	93.72	93.77
Normal	98.00	99.39	98.00	98.69	98.69
Average	**97.50**	**95.02**	**97.50**	**96.21**	**96.23**
Epoch − 1000					
Fire	99.00	92.52	99.00	95.65	95.71
Normal	98.40	99.80	98.40	99.09	99.10
Average	**98.70**	**96.16**	**98.70**	**97.37**	**97.40**
Epoch − 1500					
Fire	100.00	93.46	100.00	96.62	96.67
Normal	98.60	100.00	98.60	99.30	99.30
Average	**99.30**	**96.73**	**99.30**	**97.96**	**97.99**
Epoch − 2000					
Fire	92.00	81.42	92.00	86.38	86.55
Normal	95.80	98.36	95.80	97.06	97.07
Average	**93.90**	**89.89**	**93.90**	**91.72**	**91.81**
Epoch − 2500					
Fire	91.00	89.22	91.00	90.10	90.10
Normal	97.80	98.19	97.80	98.00	98.00
Average	**94.40**	**93.70**	**94.40**	**94.05**	**94.05**
Epoch − 3000					
Fire	98.00	91.59	98.00	94.69	94.74
Normal	98.20	99.59	98.20	98.89	98.89
Average	**98.10**	**95.59**	**98.10**	**96.79**	**96.82**

**Fig. 8:**
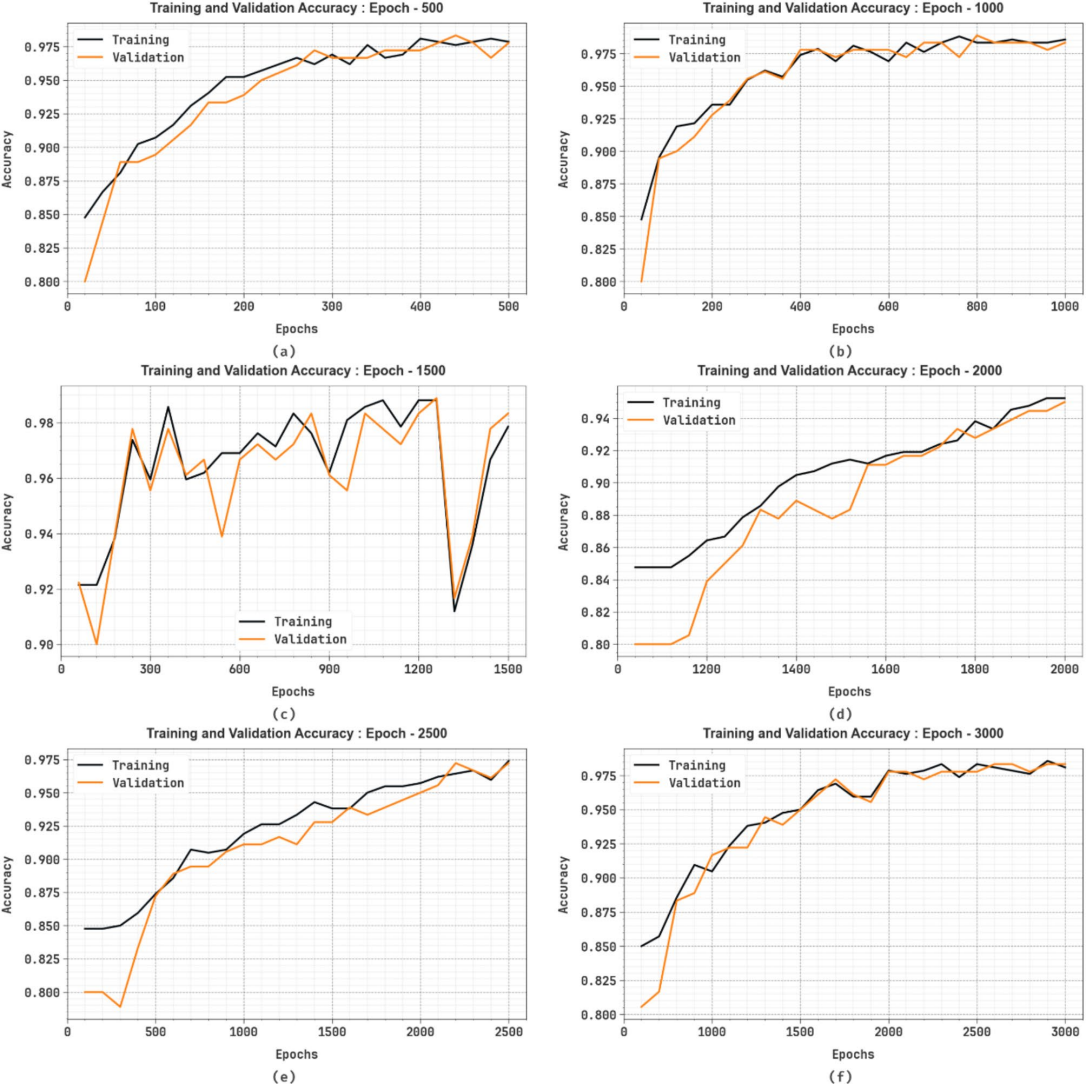
$\:Acc{u}_{y}$ graph of SFDAB-ARNNSHO methodology (**a**–**f**) Epochs 500–3000.

Figure 8 illustrates the training (TRA) $\:acc{u}_{y}$ and validation (VAL) $\:acc{u}_{y}$ outcomes of the SFDAB-ARNNSHO technique under different epochs. The $\:acc{u}_{y}\:$analysis is computed across the range of 0-3000 epochs. The figure emphasized that the TRA and VAL $\:acc{u}_{y}$ analysis displays an increasing trend, which informed the capacity of the SFDAB-ARNNSHO method to have superior outcomes across multiple iterations.

**Fig. 9:**
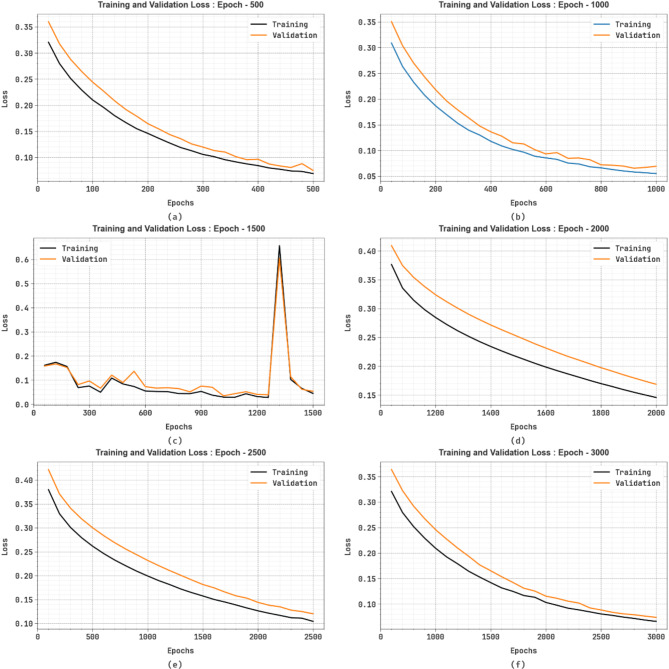
Loss analysis of SFDAB-ARNNSHO methodology (**a**–**f**) Epochs 500–3000.

Figure 9 shows the TRA loss (TRALOS) and VAL loss (VALLOS) analysis of the SFDAB-ARNNSHO approach under dissimilar epochs. The loss values are calculated across the range of 0-3000 epochs. It is denoted that the TRALOS and VALLOS values exemplify a diminishing trend, informing the SFDAB-ARNNSHO technique’s capacity to balance a trade-off between data fitting and simplification.

**Fig. 10:**
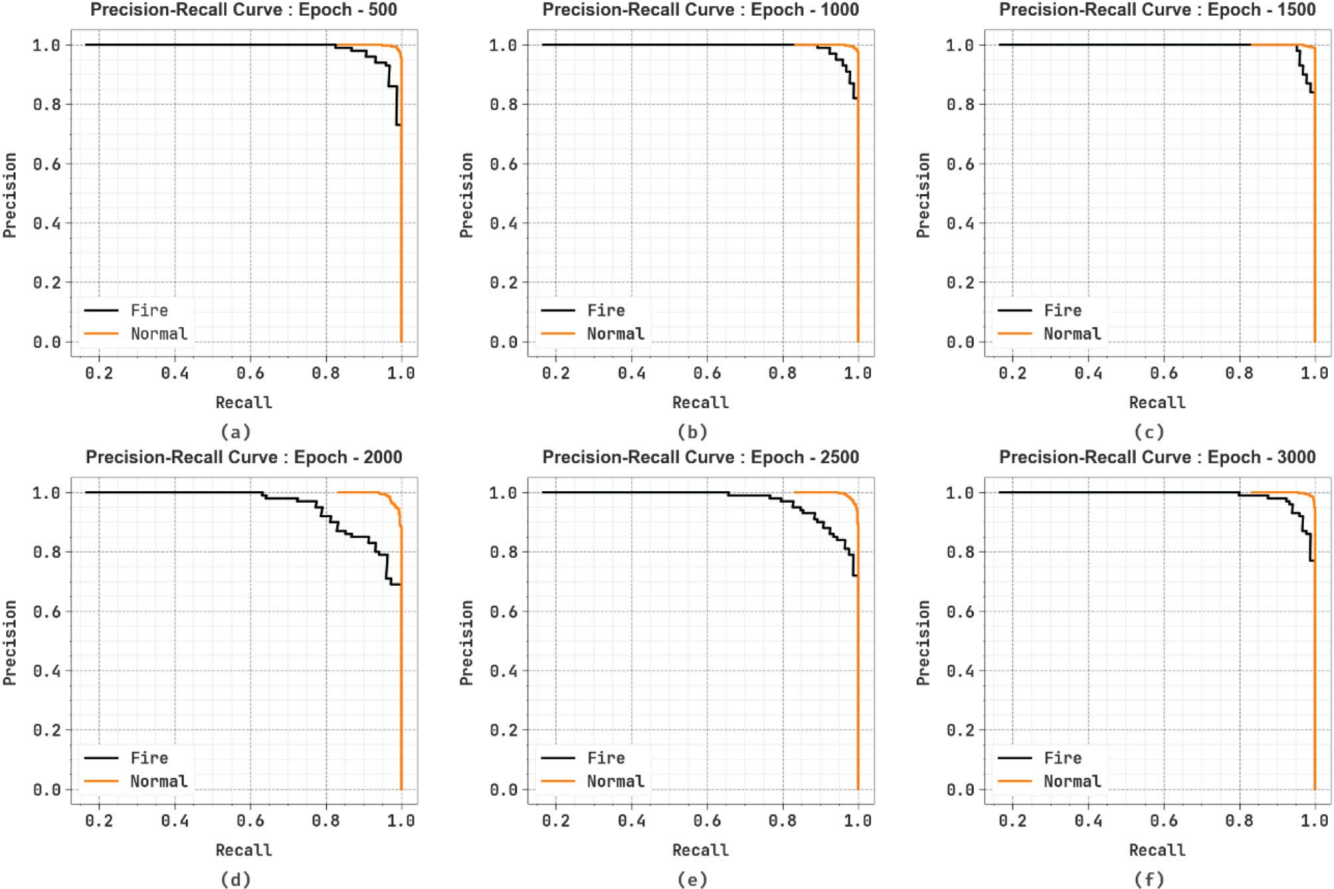
PR analysis of SFDAB-ARNNSHO method (**a**–**f**) Epochs 500–3000.

In Fig. 10, the PR graph analysis of the SFDAB-ARNNSHO methodology under different epochs clarifies its outcome by plotting Precision beside Recall for two labels. The result shows that the SFDAB-ARNNSHO technique constantly achieves better PR values than dissimilar classes. It demonstrates its size to keep a vital section of true positive predictions among all the positive predictions (precision), taking a massive ratio of real positives (recall).

**Fig. 11:**
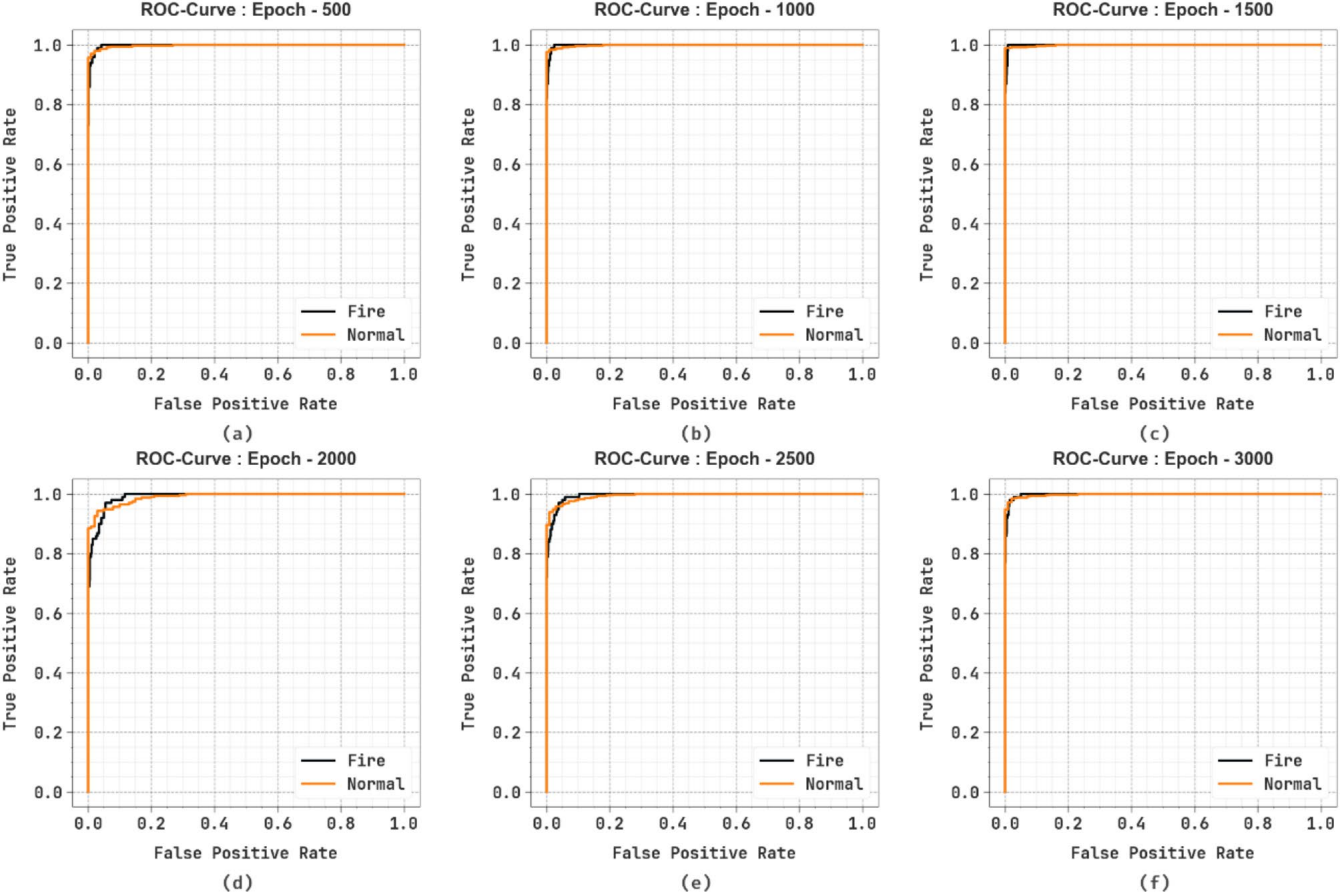
ROC graph of SFDAB-ARNNSHO approach (**a**–**f**) Epochs 500–3000.

Figure 11 examines the ROC graph of the SFDAB-ARNNSHO approach under different epochs. The results showed that the SFDAB-ARNNSHO technique gains maximum ROC across all classes, demonstrating the critical ability to discriminate the class labels. This dependable tendency of higher ROC analysis across various classes shows the capable outcome of the SFDAB-ARNNSHO methodology on predicting class labels, highlighting the robust nature of the classification method.

**Fig. 12:**
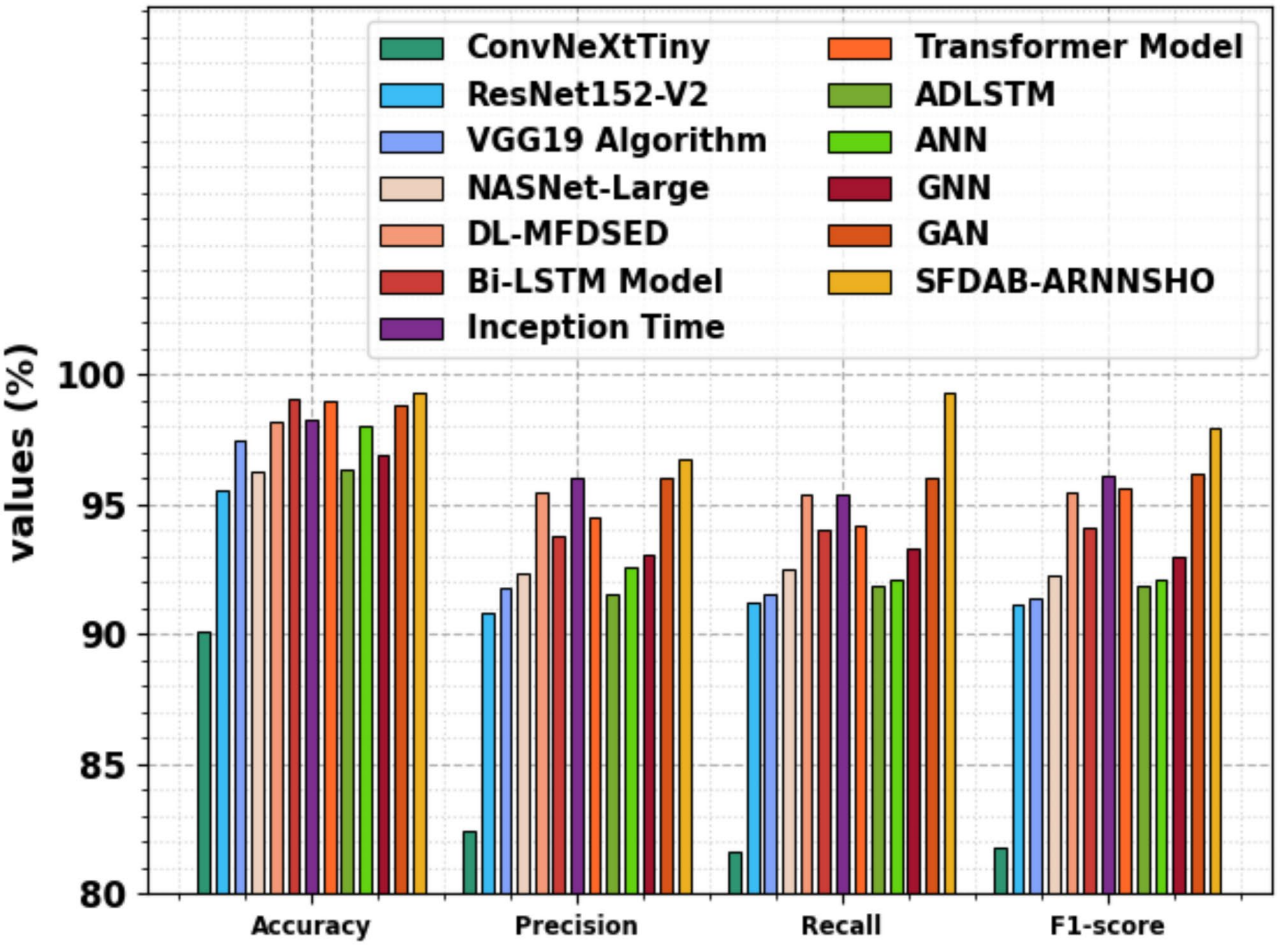
Comparative outcome of SFDAB-ARNNSHO technique with existing models.

The comparative outcome of the SFDAB-ARNNSHO model with existing methodologies is demonstrated in Table 3; Fig. 12^19,46–48^. The simulation results stated that the SFDAB-ARNNSHO approach outperformed better performances. Based on $\:acc{u}_{y}$, the SFDAB-ARNNSHO approach has a higher $\:acc{u}_{y}$ of 99.30%. In contrast, the ConvNeXtTiny, ResNet152-V2, VGG19, NASNet-Large, DL-MFDSED, Bi-LSTM, Inception Time, Transformer approaches, ADLSTM, artificial neural network (ANN), graph neural networks (GNNs), and generative adversarial network (GAN) exhibits lesser $\:acc{u}_{y}$ of 90.08%, 95.56%, 97.46%, 96.29%, 98.17%, 99.08%, 98.25%, 98.97%, 96.36%, 98.05%, 96.89%, and 98.81%, respectively. Also, depend on $\:pre{c}_{n}$, the SFDAB-ARNNSHO approach has superior $\:pre{c}_{n}$ of 96.73% where the ConvNeXtTiny, ResNet152-V2, VGG19, NASNet-Large, DL-MFDSED, Bi-LSTM, Inception Time, and Transformer techniques, ADLSTM, ANN, GNNs, and GAN illustrates minimal $\:Pre{c}_{n}$ of 82.46%, 90.84%, 91.82%, 92.34%, 95.47%, 93.79%, 96.05%, 94.53%, 91.54%, 92.58%, 93.06%, and 96.02%, correspondingly. Moreover, for $\:{the\:F1}_{score}$, the SFDAB-ARNNSHO approach has a maximal $\:{F1}_{score}$ of 97.96%. In contrast, the ConvNeXtTiny, ResNet152-V2, VGG19, NASNet-Large, DL-MFDSED, Bi-LSTM, Inception Time, Transformer techniques, ADLSTM, ANN, GNNs, and GAN depicts inferior $\:{F1}_{score}$ of 81.77%, 91.18%, 91.38%, 92.27%, 95.45%, 94.12%, 96.11%, 95.65%, 91.89%, 92.12%, 92.97%, and 96.20%, correspondingly.

**Table 3:** Comparative results of SFDAB-ARNNSHO technique with existing models^19,46–48^.

Technique	$Acc{u_y}$	$Pre{c_n}$	$Rec{a_l}$	$F{1_{score}}$
ConvNeXtTiny	90.08	82.46	81.65	81.77
ResNet152-V2	95.56	90.84	91.23	91.18
VGG19 algorithm	97.46	91.82	91.51	91.38
NASNet-large	96.29	92.34	92.52	92.27
DL-MFDSED	98.17	95.47	95.36	95.45
Bi-LSTM model	99.08	93.79	93.99	94.12
Inception time	98.25	96.05	95.37	96.11
Transformer model	98.97	94.53	94.21	95.65
ADLSTM	96.36	91.54	91.85	91.89
ANN	98.05	92.58	92.08	92.12
GNN	96.89	93.06	93.28	92.97
GAN	98.81	96.02	96.00	96.20
SFDAB-ARNNSHO	99.30	96.73	99.3	97.96

**Fig. 13:**
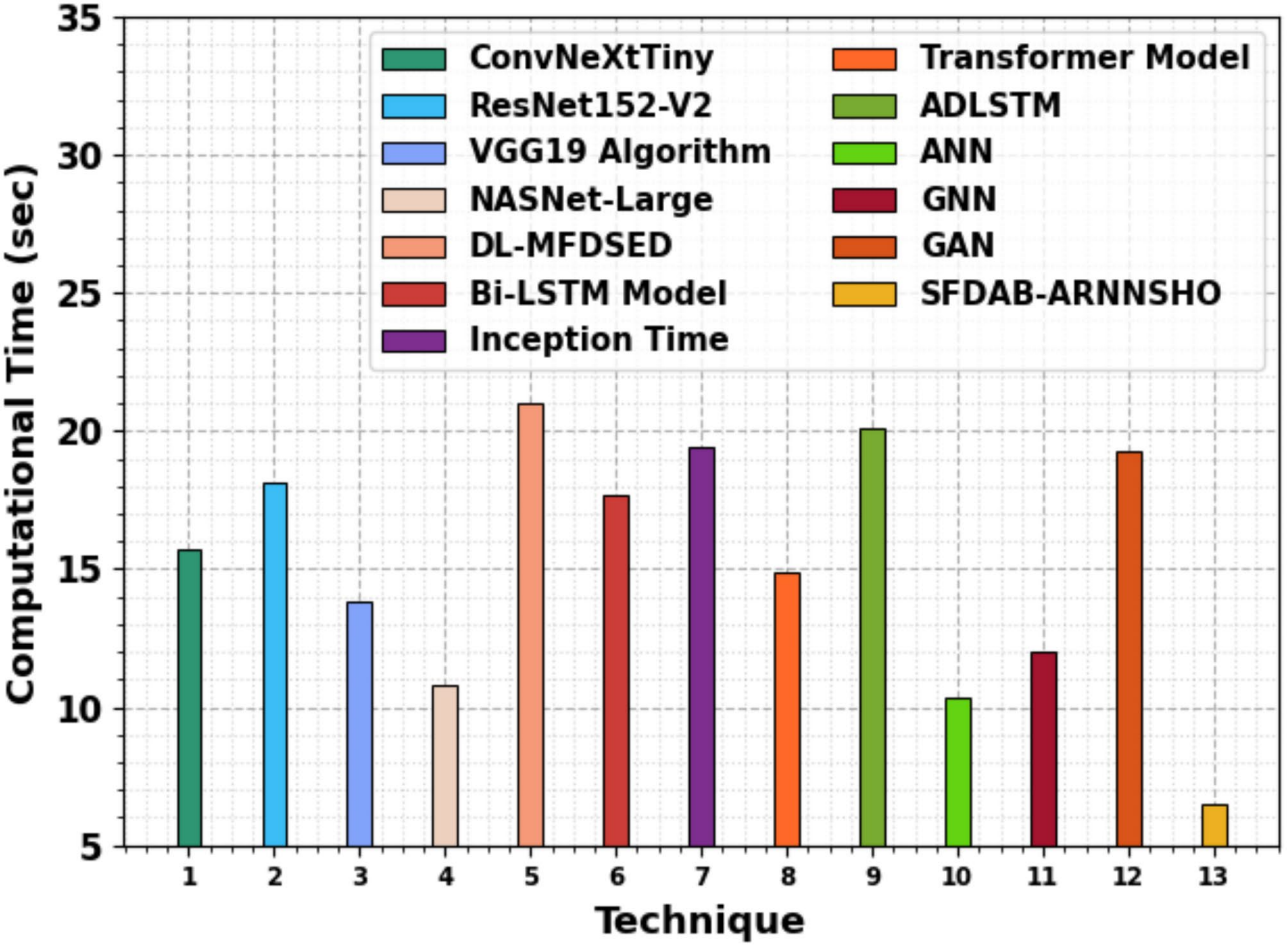
ET outcome of SFDAB-ARNNSHO approach with existing methods.

In Table 4; Fig. 13, the comparative results of the SFDAB-ARNNSHO methodology are identified in terms of execution time (ET). The table values suggest that the SFDAB-ARNNSHO approach gets a higher outcome. Depending on ET, the SFDAB-ARNNSHO approach presents a lower ET of 10.86 s. In contrast, the ConvNeXtTiny, ResNet152-V2, VGG19, NASNet-Large, DL-MFDSED, Bi-LSTM, Inception Time, and Transformer techniques, ADLSTM, ANN, GNNs, and GAN accomplish greater ET values of 16.27 s, 30.14 s, 21.03 s, 26.37 s, 18.08 s, 18.14 s, 37.83 s, 33.14 s, 20.11 s, 10.38 s, 11.98 s, and 19.30 s, correspondingly.

**Table 4:** ET outcome of SFDAB-ARNNSHO approach with existing techniques.

Technique	ET (s)
ConvNeXtTiny	16.27
ResNet152-V2	30.14
VGG19 algorithm	21.03
NASNet-large	26.37
DL-MFDSED	18.08
Bi-LSTM model	18.14
Inception time	37.83
Transformer model	33.14
ADLSTM	20.11
ANN	10.38
GNN	11.98
GAN	19.30
SFDAB-ARNNSHO	10.86

## Conclusion

In this manuscript, a novel SFDAB-ARNNSHO method is presented. The main intention of the SFDAB-ARNNSHO method is to detect and classify fire for blind people. To achieve this, the proposed SFDAB-ARNNSHO model involves image pre-processing stage SF to remove noise in input data. Furthermore, the fusion of feature extraction adopts three methods: EfficientNetB7, CapsNet, and ShuffleNetV2. In addition, the SFDAB-ARNNSHO model performs fire detection and classification using the SBiLSTM-AM technique. Finally, the parameter tuning of the SBiLSTM-AM method is executed by the design of the SHO method. The simulation validation of the SFDAB-ARNNSHO methodology is examined under the fire detection dataset, and the outcomes are measured using various measures. The performance validation of the SFDAB-ARNNSHO methodology portrayed a superior accuracy value of 99.30% over existing models under diverse measures. The limitations of the presented study SFDAB-ARNNSHO methodology comprise challenges related to the robustness of fire detection in highly dynamic or noisy environments, where environmental factors such as lighting changes or smoke density may affect performance. Additionally, the model’s reliance on high-quality image data could pose challenges in real-world scenarios where data quality may vary. Another limitation is the computational complexity, which may impact real-time implementation on resource-constrained devices. The technique’s capability to generalize across diverse fire scenarios and environments remains an area for enhancement. Future works can improve model robustness to varying conditions, optimize it for faster processing, and explore integrating additional sensor data (e.g., thermal imaging) to improve detection accuracy. Further research can also explore the model’s scalability for broader applications, including integration with IoT devices for real-time monitoring and response.

## Data Availability

The data that support the findings of this study are openly available in the Kaggle repository at https://www.kaggle.com/datasets/atulyakumar98/test-dataset.“.
